# Learnability Advantage of Segmental Repetitions in Word Learning

**DOI:** 10.1177/00238309231223909

**Published:** 2024-02-05

**Authors:** Johanna Basnak, Mitsuhiko Ota

**Affiliations:** School of Philosophy, Psychology & Language Sciences, The University of Edinburgh, UK

**Keywords:** Domain-general biases, wordform learning, repetitions, lexical processing, consonants and vowels

## Abstract

To date, research on wordform learning biases has mostly focused on language-dependent factors, such as the phonotactics and neighborhood density of the language(s) known by the learner. Domain-general biases, by contrast, have received little attention. In this study, we focus on one such bias—an advantage for string-internal repetitions—and examine its effects on wordform learning. Importantly, we consider whether any type of segmental repetition is equally beneficial for word recall, or whether learning is favored more or only by repeated consonants, in line with previous research indicating that consonants play a larger role than vowels in lexical processing. In Experiment 1, adult English speakers learned artificial consonant-vowel-consonant-vowel words containing either a repeated consonant (e.g., /sesu/, “c-rep”), a repeated vowel (e.g., /sepe/, “v-rep”), or dissimilar consonants and vowels (e.g., /sepu/, “no-rep”). Recall results showed no advantage for v-reps but higher accuracy for c-reps compared with no-reps. In Experiment 2, participants performed a label preference task with the same stimuli. The results showed dispreference for both c-reps and v-reps relative to no-reps, indicating that the results of Experiment 1 are independent of wordlikeness effects. These outcomes reveal that there is a form-learning bias for words with identical consonants but not for words with identical vowels, suggesting that a domain-general advantage for repetitions within strings is modulated by a language-specific processing bias for consonants.

## 1 Introduction

### 1.1 Language-dependent and -independent biases in wordform learning

When learners encounter a novel word, they need to create a new memory representation for its phonological form or wordform. During this process of wordform learning, learners can refer to at least two types of existing knowledge: their knowledge of the typical sound patterns of the language (e.g., phonotactics or the occurrence probability of individual sounds and sound combinations) and their knowledge of other words in the language’s lexicon (e.g., neighborhood density or the number of words that sound similar to a given word). A large body of work in the past few decades has demonstrated that both types of knowledge—that is, phonological and lexical—influence the process by which new wordforms are learned. Regarding phonological knowledge, phonotactics have been repeatedly shown to affect how easily nonwords are recognized, recalled, and mapped onto novel objects (e.g., González-Gómez et al., 2013; Graf Estes et al., 2011; MacKenzie et al., 2012; Storkel, 2001, 2003; Storkel et al., 2006; Storkel & Lee, 2011). In relation to lexical knowledge, novel words with dense neighborhoods have advantages in short-term memory storage (Edwards et al., 2004; Metsala & Chisholm, 2010; Roodenrys & Hinton, 2002; Thomson et al., 2005), the development of early lexical entries (Hollich et al., 2002; S. E. Jones, 2018; Newman et al., 2008; Stamer & Vitevitch, 2012; Storkel, 2001, 2003, 2004, 2009), and their integration in the developing lexicon (Storkel & Lee, 2011). These findings show that learning new words critically involves knowledge that is specific to the sound patterns and the lexicon of the language.

However, wordform learning may also be guided by general learning biases independent of the particular phonological or lexical patterning of the language(s) known to the learner. Although such type of learning bias has received much less attention compared with that of language-dependent phonological and lexical information, the potential role it plays in word learning is important for three reasons. First, these language-independent factors may underlie some of the phonotactic effects that are usually attributed to the specific language that is studied. For example, if certain sound combinations are more frequent across languages, at least some of the correlations between phonotactic probabilities and wordform learning could be due to an inherent learnability of words with those common sound combinations rather than their frequency in the given language. Understanding general biases in wordform learning will allow us to investigate whether such an interaction occurs between language-dependent and language-independent effects and, if so, to disentangle the two types of sources.

A second point, related to the first, is that the presence of language-independent biases in learning wordforms can shed light on general tendencies in the sound patterns of the world’s languages. Cross-linguistically common sound combinations may be a product of biased diachronic selection of lexical items that feature those combinations (Martin, 2007; Matzinger & Ritt, 2022). If lexical forms with certain phonological characteristics are found to be easier to learn than others, they may be preferentially retained during language change and transmission, eventually resulting in a phonotactic skew that is shared among many languages. Thus, by examining language-independent biases in learning wordforms, we may be able to identify lexical factors that contribute to typological tendencies in phonological patterns.

Third, language-independent biases in wordform learning may offer a window on the effects of domain-general cognitive biases on language learning. One established language-independent effect on wordform learning is the advantage that shorter words have in immediate repetition tasks (e.g., Gathercole & Baddeley, 1989; Gathercole et al., 1992; Girbau & Schwartz, 2007; Gupta, 2003; Papagno & Vallar, 1992; Radeborg et al., 2006; Szewczyk et al., 2018; Topbaş et al., 2014). This effect of length in recall accuracy must stem from general constraints on processing and memory since it is also observed in tasks involving nonlinguistic stimuli—such as sequences of digits (G. Jones & Macken, 2015) or dots appearing in different spatial locations (D. Jones et al., 1995)—and in equivalent experiments performed by nonhuman subjects (e.g., macaques: Botvinick et al., 2009). Other language-independent effects in wordform learning may also be grounded in domain-general biases and can contribute to our understanding of the link between general cognitive mechanisms and language learning.

### 1.2 Repetitions in wordform learning

With these points in the backdrop, in this study, we have elected to address the role of sound repetitions within a word as a potential source of general bias in wordform learning. A domain-general foundation for this learning bias can be easily found in studies using nonverbal stimuli. For instance, in serial recall tasks of letter sequences, identical letters occurring close together are more accurately recalled than two different letters in corresponding positions (Crowder, 1968; Henson, 1998). In experiments involving musical tones, sequences containing repeated tones are easier to learn (Endress et al., 2007; Monaghan & Rowson, 2008). Similar effects have been observed in sequences of objects varying in shape, color, and size and are generally attributed to compressibility of information encoding repeated elements in strings (Chekaf et al., 2016; Feldman, 2000).

There is some indication that word learning is also subject to a similar facilitatory effect of sound repetitions. For example, English-learning 7-month-olds are better at segmenting and remembering novel wordforms that contain identical vowels (as well as wordforms containing vowels agreeing in backness and rounding) than those that do not (Mintz et al., 2018). Relatedly, compared with novel words with no sound repetitions (e.g., *nifu*), those consisting of repeated syllables (e.g., *nini*) are more easily segmented from continuous speech by English-learning infants at 9 months (Ota & Skarabela, 2018), and better learned as labels of unfamiliar referents at 18 months (Ota & Skarabela, 2016). Furthermore, adult English speakers recall novel words with repetitions of consonants (e.g., *wi* ***k*** *o* ***k*** *a*) more accurately than those without segmental repetitions (e.g., *kilepo*) (Ota et al., 2021).

Although these results are consistent with the existence of a language-independent learning bias for wordforms with sound repetitions, it is difficult to draw definite conclusions from them for a number of reasons. First, the aforementioned studies were all conducted on speakers or learners of English, raising the question of whether the observed learning bias could at least partly stem from certain characteristics pertaining to the English language. Second, there is a vast amount of heterogeneity in the studies. Some of these studies (e.g., Mintz et al., 2018; Ota & Skarabela, 2018) are concerned with word segmentation (i.e., how word like units are identified in running speech) rather than word learning (i.e., how wordforms are remembered). The studies also differ in participant age, ranging from 7-month-olds to adults. Third, the generalizability of the findings is limited by methodological constraints. In particular, the experiment in Ota et al. (2021) was carried out with orthographic words containing sound repetitions that varied incidentally, and it remains to be seen whether the results can be replicated with spoken words that are systematically controlled for internal repetitions. Finally, none of the studies, except Ota et al. (2021), was designed to compare the repetition effects attributable to different sound units. Notwithstanding the methodological issues mentioned just above, the Ota et al.’s study showed that novel word recall was facilitated by repetitions of consonant letters but not by repetitions of vowel letters. It is possible therefore that the effects of word-internal sound repetition differ by segment type, with consonants and vowels exhibiting different behaviors.

The possibility that consonants and vowels may contribute differently to string-internal repetition effects on wordform learning is in alignment with the notion that consonants play a larger role than vowels in marking lexical distinctions (see Nazzi & Cutler, 2019 for a review). A key source of evidence in support of the advantage conferred by consonants in lexical processing is research showing that learners and speakers—from toddlers to adults—preferentially attend to consonants rather than vowels in word learning and other lexical tasks, such as word recognition and word reconstruction. This effect has been reported for a range of languages, including English (Creel et al., 2006; Nazzi et al., 2009), French (e.g., Havy et al., 2014; Havy & Nazzi, 2009), Dutch (Cutler et al., 2000), Japanese (Cutler & Otake, 2002), Sesotho (Cutler et al., 2002), and Slovak (Hanulíková et al., 2011). If consonants generally carry more weight than vowels in encoding lexical contrasts, we might also expect the bias to extend to the effect of sound repetitions, such that facilitation in learning from repetition is unique to consonants, or larger in consonants than vowels.

A general learning bias for repeated consonants may have implications not only for word learning but also for the typological properties of the phonological lexicon in natural languages. One such property is what is known as the “identity effect” (Gallagher, 2013; MacEachern, 1999). The identity effect is a counter-effect to a wide range of phonological constraints on the cooccurrence of consonants that share a feature such as major place, aspiration, and ejective. In many languages, such cooccurrence restrictions fail to apply, or underapply, to identical segments. For example, in languages such as Muna, Javanese, and Aymara, words containing two nonidentical consonants sharing a major place of articulation (e.g., /m. . .p/) are underrepresented, but words containing identical consonants (e.g., /p. . .p/) are not, and are even overrepresented in some cases (Coetzee & Pater, 2008; Graff & Jaeger, 2009). The identity effect would be congruent with, and perhaps partly explained by, a learning bias for words with identical or repeated consonants. We return to this issue, and other implications of a consonant repetition bias in word learning, in the General Discussion.

### 1.3 Purpose of this study

In sum, previous work suggests that wordform learning may be subject to a general bias for word-internal sound repetitions. Such an effect is likely to be rooted in a domain-general learning bias for string-internal unit repetitions, but also modulated by a language-specific processing bias for consonants. This is consistent with the vast literature showing that speakers and learners of typologically different languages favor consonants in a range of lexical processing tasks, as well as with Ota et al.’s (2021) finding that wordform learning is facilitated only from repetitions of consonants in contrast to repetitions of vowels. However, conclusive evidence is lacking as no studies have investigated this question by directly comparing the effects of consonant and vowel repetitions in wordform learning using spoken word stimuli and independently manipulating the occurrence of word-internal segmental repetitions.

The purpose of the present study was to address this research gap by systematically testing whether the repetition of consonants facilitates word learning, and if so, to a greater extent than the repetition of vowels. Adult English speakers were familiarized with novel consonant-vowel-consonant-vowel (CVCV) words, some with two identical consonants but different vowels (C_1_V_1_C_1_V_2_), some with two identical vowels but different consonants (C_1_V_1_C_2_V_1_), and some without any identical consonants or vowels (C_1_V_1_C_2_V_2_). The participants were subsequently tested on their recall of these words. We predicted that words with consonant repetitions should be more accurately recalled than those with vowel repetitions or no segmental repetitions. To dissociate this contrast from differences related to English-specific phonological and lexical properties, the novel words were pre-experimentally controlled for phonotactic probabilities and neighborhood statistics. An additional experiment was carried out to rule out the possibility that the effects from the main experiment are due to perceived differences in the wellformedness or wordlikeness of the novel words.

## 2 Experiment 1: recall task

### 2.1 Method

In this experiment, we tested adults’ learning of auditorily presented novel wordforms with consonant repetitions, vowel repetitions, or dissimilar consonants and vowels. Participants were asked to remember associations between the novel words and unfamiliar objects. Two tests were given: an object-matching test in which participants selected the object that matched each word, and a recall test where they verbally reproduced, from memory, the wordform (or label) for each object. Only the recall test was the subject of analysis: the variable of interest was production accuracy, measured as the edit distance between the target form and the recalled form. The experiment was run online using the platform Testable (Rezlescu et al., 2020), which participants accessed on a desktop computer via a weblink.

#### 2.1.1 Participants

Participants, recruited via Prolific (www.prolific.co), were 72 adult native speakers of English who had an approval rate of 95% or higher and a minimum of 50 previous submissions to the online platform. There were 55 females, 16 males, and one person who preferred not to indicate their sex, ranging in age from 19 to 60 years (*M* = 34.2 years). All participants were self-reported monolingual English speakers, born in one of five English-speaking countries: U.K. (*n* = 55), U.S.A. (*n* = 13), Canada (*n* = 2), Australia (*n* = 1), and Ireland (*n* = 1). Most of them were residents in their country of origin, except for three U.S. nationals currently living in the U.K., and three U.K.-born participants residing in Australia (*n* = 1), Ireland (*n* = 1), and Germany (*n* = 1).

Participants were asked to list any foreign languages they had studied. These included French (*n* = 30), German (*n* = 21), Spanish (*n* = 18), Italian (*n* = 6), Irish (*n* = 2), Ancient Greek (*n* = 1), Arabic (*n* = 1), Esperanto (*n* = 1), Japanese (*n* = 1), Korean (*n* = 1), Latin (*n* = 1), Polish (*n* = 1), Russian (*n* = 1), and Welsh (*n* = 1). A compensation of £4 was paid for completing the experiment, which on average took 15 min and 40 s. An additional five participants took part in the study, but their data were not included in the analysis as they did not fully follow the instructions, leading to an incomplete recording of their responses.

#### 2.1.2 Materials

##### 2.1.2.1 Unfamiliar objects

The visual prompts presented to participants were photographs of 36 unfamiliar objects from the Novel Object and Unusual Name (NOUN) database (Horst & Hout, 2016). Half of these were identical to those used in Ota et al. (2021). The other 18 were selected on the basis of their relatively low familiarity scores (the percentages of people who indicated that they had seen a similar picture before) and nameability scores (the percentages of people who could spontaneously come up with a name for the object). The mean familiarity score for the 36 pictures was 21.8 (*SD* = 12.9), and the mean nameability score was 36.7 (*SD* = 13.2). The photographs are listed in Appendix A.

##### 2.1.2.2 Novel words

The critical stimuli consisted of 24 triplets of novel CVCV words, each of them comprising one item with a consonantal repetition (C_1_V_1_C_1_V_2_; e.g., *fifo*, hereafter “c-rep”), one with a vocalic repetition (C_1_V_1_C_2_V_1_; e.g., *fiti*, hereafter “v-rep”), and one with no segmental repetitions, comprising dissimilar consonants and vowels (C_1_V_1_C_2_V_2_; e.g., *fito*, hereafter “no-rep”).^1^ The c-rep word differed from the no-rep word only by the second consonant, and the v-rep word differed from the no-rep word only by the second vowel. To maximize the phonological distance between segments in no-rep items, we combined consonants and vowels in each word such that the two vowels differed in height and backness (e.g., /i-o/, /u-e/, /e-a/) and the two consonants differed both in place and manner of articulation (e.g., /k-f/, /s-p/, /t-m/). Voicing was not manipulated in no-rep words, where obstruents were always voiceless, as consonants with different places and manners were considered to be sufficiently different from each other for the purpose of this study.

Table 1 presents key English statistics for the neighborhood density and phonotactics of the critical novel words, which were compiled using the Irvine Phonotactic Online Dictionary (IPhOD) version 2 (Vaden et al., 2009). Neighborhood density was calculated as the number of real English words in the IPhOD database that differed from the novel word by a single segment addition, deletion, or substitution. Positional segment probability was calculated as the average probability of the segment occurring in the specific position among English words that contain the same number of segments (i.e., 4). Biphone and triphone probability were calculated as the average probability of two- and three-segment combinations occurring in English words. The probability measures were log-transformed. There was no statistical difference between c-reps, v-reps, and no-reps for any of these measures. Care was also taken to avoid items that could sound familiar to English speakers either because they resemble an English word (e.g., *siso, pilo*), they correspond to a brand name (e.g., *fila*), or they are very common Spanish words (e.g., *papi*; as explained below, the words were synthesized using Spanish as a source language). Prior to the experiment, the critical items were submitted to a familiarity rating task taken by 7 native English speakers (3 females, 4 males; mean age = 46) who did not take part in Experiment 1 and were asked to rank each word on a scale of 1 to 9 according to how familiar it sounded. No statistical difference was found between the mean ratings given to c-reps, v-reps, and no-reps (see the last rows in Table 1).

**Table 1.** Lexical Characteristics of Critical Items.

Measure	C-reps	V-reps	No-reps	*F*(2, 69)	*p*
Neighborhood density					
*M*	2.9	3.6	3.3	.291	.748
*SD*	3.0	4.0	3.8		
Positional segment probability (log)					
*M*	0.047	0.044	0.045	.212	.810
*SD*	0.014	0.016	0.015		
Biphone probability (log)					
*M*	0.0012	0.0011	0.0011	0.120	0.887
*SD*	0.0008	0.0008	0.0008		
Triphone probability (log)					
*M*	1.4e-05	1.6e-05	1.6e-05	0.252	0.778
*SD*	1.6e-05	2.2e-05	1.8e-05		
Familiarity rating (scale: 1–9)					
*M*	3.7	3.6	3.3	.141	.869
*SD*	1.2	1.3	1.1		

In addition to the critical items, there were 12 filler items, half of them CVCVC words (e.g., *nepas*) and the other half, CVCVCV words (e.g., *pisole*). The fillers had different syllable structures from the critical items to minimize potential learning interference with the critical items. All novel words, both critical items and fillers, are listed in Appendix B.

The stimuli were auditorily rendered with the speech synthesizer Wideo (https://wideo.co/text-to-speech/) using the voice of a male Spanish speaker. Spanish was selected as it has a straightforward five-vowel system that exhibits little reduction in unstressed syllables. The segments used were /i, e, u, o, a, p, t, k, f, s, ʧ, l, m, n/, all of which have clear correspondents in English and none with obvious allophonic variants (e.g., voiced stops in Spanish undergo spirantization intervocalically). The stimuli were normalized at an amplitude of 70 dB and were approximately 600 ms in duration.

As the main measure of this experiment was recall accuracy in the reproduction of these words, we checked participants’ perception of the synthesized stimuli as a precondition for learning. Ten native speakers of English (7 females, 3 males; mean age = 33 years), recruited on Prolific and who did not take part in Experiment 1, were asked to repeat each of the critical stimuli immediately after hearing them. The responses were transcribed independently by the two authors using broad phonetic symbols (ARPABET). There was 98.4% agreement at the segment level between the two sets of transcription. When there were disagreements between the transcriptions, the one that was closer to the target was selected. Although some English speakers produced somewhat diphthongized versions of the monophthongal Spanish mid-vowels /e/ and /o/, none of the 1,431 vowels in participants’ productions were deemed to be anything other than the five canonical vowels /a, i, u, e, o/. A large proportion of the differences between the intended synthesis and participants’ responses was due to a mismatch in voicing (40%; e.g., *sopek* returned as [sobek]) or affrication (18%; e.g., *kuʧu* returned as [kuʃu]). This is most likely due to differences in voice onset time (VOT) between Spanish (the source language of the synthesis) and English (the participants’ native language). Spanish voiceless stops are typically produced with a short-lag VOT and could be perceived as voiced stops by speakers of English, which has a short-lag VOT for voiced stops. Similarly, affricates in Spanish could be perceived as fricatives by speakers of English, which has a more audible postrelease aspiration in affricates. We have therefore adjusted for these errors by accepting voiced stops produced for intended voiceless stops of the same place (e.g., [b] for /p/) and voiceless fricatives produced for intended affricates of the same place (e.g., [ʃ] for /ʧ/). The adjusted error rates, given in Table 2, did not differ statistically by word type. The same adjustments were made in the scoring of the main experiment, as explained later.

**Table 2.** Perception Error Rates of Critical Items.

	C-reps	V-reps	No-reps	*F*(2, 18)	*p*
*M*	0.035	0.056	0.048	1.874	.182
*SD*	0.038	0.041	0.040		

#### 2.1.3 Procedure

The novel words were organized in six training lexicons of 18 items, presented in Appendix B. Each comprised 12 critical items—equally divided between c-reps, v-reps, and no-reps—and 6 fillers, of which 3 had a CVCVC structure and 3 had a CVCVCV structure. The lexicons were counterbalanced so that none included more than one item of a triplet: for instance, the set *kika* (c-rep)—*kifi* (v-rep)—*kifa* (norep) was distributed over lexicon *F* (*kika*), lexicon C (*kifi*), and lexicon B (*kifa*). This was done to minimize interference between these minimally contrastive items.

The novel words were randomly mapped to the unfamiliar objects, generating six stimulus lists to which participants were evenly assigned. The experiment consisted of two blocks of passive training, each followed by an object-matching test for the items in the block and a short recall test with four items (one of each word type: no-rep, c-rep, v-rep, and filler). After this, a final object-matching test presented all 18 trained items once more. At the end, participants were tested on their recall through an oral production task. The details of these tasks are given below.

##### 2.1.3.1 Passive training phase

During each of the two blocks of passive training, participants were taught nine object-word mappings, where six words corresponded to critical items—two of each type—and the remaining three, to fillers. All items were block-randomized. The word-object pairs were presented through short videos consisting of a static image of the object (approximately 350 × 350 pixels in size) accompanied by the auditory stimulus. Participants were instructed to watch and learn, and could replay each video as often as they wished before moving on to the next one.

##### 2.1.3.2 Object-matching training phase

Each block of passive training was followed by an object-matching test for the items of the block. This consisted of nine trials in which four objects were shown while a word was played. Participants had to select the object corresponding to the word they heard. After each trial, they were given feedback (correct/incorrect) and the word in question was played again, while the correct image was displayed on screen. A general object-matching test, following the same procedure but comprising all 18 trained items, was presented before the final recall test.

##### 2.1.3.3 Recall phase

At the end of each training block, following the 9-item object-matching test, participants completed a short recall task involving one item of each type of word (c-rep, v-rep, no-rep, and filler). In four randomized trials, they were presented with an object and asked to say its name, preceded by the carrier phrase “This is called ___.” These were deemed practice trials for the final test and were not included in the analysis. The analysis was based on the results of the final test, in which participants had to produce the names of all 18 items. Their production was captured through the microphone on their device, which was voice-activated, and recorded at a sample rate of 44.1 kHz.

The production data were transcribed independently by the two authors using ARPABET symbols. The segment-level agreement rate between the two was 93%. When there was a discrepancy between the two transcriptions of a target but one of them matched it, the response was credited with the target-like value. Similarly, the response was credited with the target-like value in cases of discrepancy between target and transcription regarding affrication or voicing differences—such as when target /ʧ/ was transcribed as /ʃ/ or /ʒ/, or target /p/ rendered as /b/ (see the “Novel Words” section above for the justification of this decision). Finally, if there was a disagreement between the raters but neither of their transcriptions matched the target even with the allowance for affrication and voicing differences described above, one of the two transcriptions was randomly chosen.

### 2.2 Results

Analyses were carried out on 860 observations of critical items, after removing 4 responses (0.46% of the total) that were partially or fully inaudible due to delayed microphone activation. Recall accuracy was analyzed in two different ways. First, we looked at the overall accuracy of participants’ recall of the critical items. If a general learning bias for string-internal repetitions is modulated by a domain-specific lexical processing advantage of consonants over vowels, we expect participants to be more accurate in learning words with identical consonants (c-reps) than words with identical vowels (v-reps) and words with dissimilar consonants and vowels (no-reps). Second, we separately analyzed the recall accuracy of the consonants and the vowels in the critical items. If the advantage of consonant repetitions comes from the larger role that consonants play in lexical distinction, the benefits of repetition should be manifested in the retention of the consonants themselves. We therefore predict recall of consonants to be more accurate in c-reps than in v-reps or no-reps, but we do not expect recall of vowels to differ by word type.

**Figure 1.**
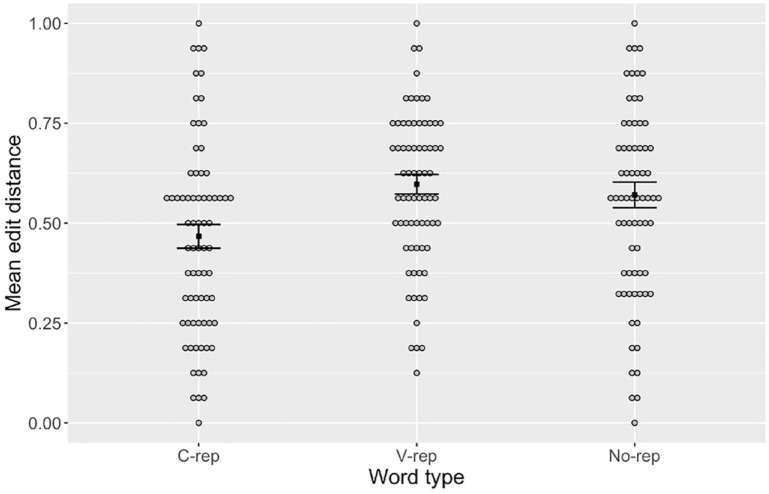
Mean Error in the Production of Words with Consonant Repetitions (“C-rep”), Vowel Repetitions (“V-rep”), and no Repetitions (“No-rep”). *Note.* Gray circles represent individual means. Black squares represent group means for each condition. Error bars are + 1 standard error of the mean.

To determine overall recall accuracy, we used Levenshtein distance, a measure employed in a range of studies on language evolution, word learning, and word production to quantify the phonological difference between a target word and its derived form (e.g., Atkinson et al., 2018; Faes et al., 2022; Kirby et al., 2008; Kurdziel & Spencer, 2016; Mulder et al., 2019; van de Ven et al., 2019). We calculated the normalized Levenshtein distance between the target word and a participant’s production of it by first taking the minimum number of segmental edits required to turn the former into the latter through insertion, deletion, or substitution. For example, if the target word was /fofi/ and the participant’s recall of that word was /fetip/, the Levenshtein distance would include two substitutions and one addition, so the edit distance is 3. This was then divided by the number of segments in the target, to give a number that roughly translates to the average number of errors per segment or the average portion of the word that was misrecalled (3/4 or 0.75 in the example above). Being an error measure, a lower edit distance indicates a better performance: the normalized edit distance of a perfect match (e.g., /fofi/ for /fofi/) is 0.0, a complete mismatch (e.g., /teka/ for /fofi/) is 1.0, and half a match (e.g., /fonu/ for /fofi/) is 0.5.^2^ Figure 1 shows the mean normalized edit distance in the production of each type of label: c-reps, v-reps, and no-reps. Despite the substantial amount of individual variation in overall accuracy level, the majority of participants (58.3%) had a lower mean edit distance for c-reps than for no-reps, and even more participants (63.9%) had a lower edit distance for c-reps than for v-reps.

We conducted a linear mixed-effects analysis on the edit distance data for c-reps, v-reps, and no-reps, with word type as a fixed effect and participant and item as random effects. The reference category for word type was set to c-reps and dummy coded for v-reps and no-reps. The intercept is therefore the model prediction for the edit distance under the c-rep condition. Analysis was conducted in R (R Core Team, 2022), using the package lme4 (Bates et al., 2015). To allay concerns that the results are affected by the perceptual accuracy of the stimulus words, we also added per-item perceptual error rates as a fixed effect. This was calculated as the mean Levenshtein distance between the intended synthesized form and the responses produced by the participants in the immediate recall task described in the “Materials” section. In the model, this variable was scaled over the entire dataset. As the full model that included random slopes resulted in a singular fit, we report the results of the model whose random effects included participant and item intercepts only.^3^ The results, presented in Table 3, showed a main effect of word type, with v-reps and no-reps significantly higher in edit distance (i.e., less accurate) than c-reps. No effect was found for perceptual error.

**Table 3.** Fixed Factor Coefficients for Edit Distance in the Production of Words.

	β	*SE*	*df*	*t*	*p*
Intercept (word type: c-rep)	0.473	0.038	105.165	12.238	<.001***
Perceptual error	0.024	0.019	70.784	1.250	.216
Word type: no-rep	0.099	0.046	68.576	2.172	.033*
Word type: v-rep	0.020	0.046	68.667	2.582	.012*

*** *p* < .001; *p < .05.

**Figure 2.**
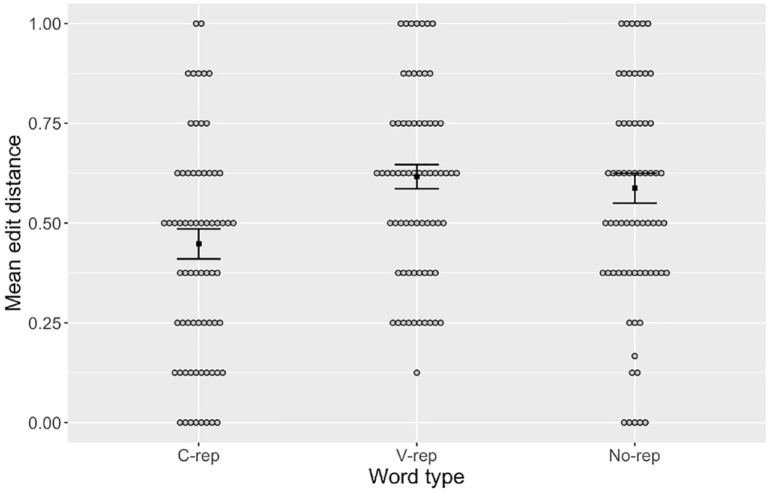
Mean Edit Distance Between Targets and Recalled Productions of Consonants in Words with Consonant Repetitions (“C-rep”), Vowel Repetitions (“V-rep”), and No Repetitions (“No-rep”). *Note.* Gray circles represent individual means. Black squares represent group means for each condition. Error bars are + 1 standard error of the mean.

**Figure 3.**
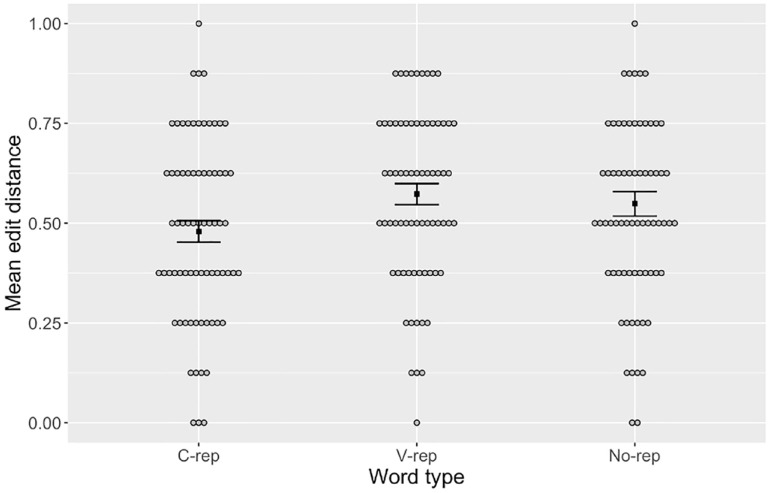
Mean Edit Distance Between Targets and Recalled Productions of Vowels in Words with Consonant Repetitions (“C-rep”), Vowel Repetitions (“V-rep”), and No Repetitions (“No-rep”). *Note.* Gray circles represent individual means. Black squares represent group means for each condition. Error bars are + 1 standard error of the mean.

To assess whether the overall accuracy for a given word type was contingent on a better retention of the consonants or vowels within participants’ productions, we computed the normalized Levenshtein distance between the target and response for consonants and vowels separately in each type of label. For example, if a participant’s response for the target word /foti/ was /fepa/, the consonant-only analysis measured the normalized edit distance between /f_t_/ and /f_p_/ (a distance of 1/2 = 0.5) and the vowel-only analysis measured that between /_o_i/ and /_e_a/ (a distance of 2/2 = 1.0). These are illustrated in Figures 2 and 3, respectively. The results were submitted to linear mixed models (see Tables 4 and 5) with word type and perceptual errors as fixed effects and participant and item as random effects. As with the previous analysis, word type was referenced to c-rep and dummy coded for no-rep and v-rep, whereas perceptual error was scaled to the entire dataset. Random slopes were removed to avoid singularity.^4^ The results showed significant effects for perceptual errors but also for no-rep and v-rep, indicating that mean error in the production of consonants was lower in c-reps than in v-reps and no-reps. By contrast, no significant effect of word type was found in the production of vowels.

**Table 4.** Fixed Factor Coefficients for Edit Distance in the Production of Consonants.

	β	*SE*	*df*	*t*	*p*
Intercept (word type: c-rep)	0.461	0.046	105.245	10.060	<.001***
Perceptual error	0.053	0.023	70.937	2.341	.022*
Word type: no-rep	0.129	0.054	68.730	2.383	.020*
Word type: v-rep	0.144	0.055	68.824	2.6441	.001*

*** *p* < .001; * *p* < .05.

**Table 5.** Fixed Factor Coefficients for Edit Distance in the Production of Vowels.

	β	*SE*	*df*	*t*	*p*
Intercept (word type: c-rep)	0.477	0.039	87.580	12.236	<.001***
Perceptual error	−0.008	0.021	71.711	0.372	.711
Word type: no-rep	0.073	0.050	69.612	1.452	.151
Word type: v-rep	0.097	0.050	69.694	1.911	.060

*** *p* < .001.

### 2.3 Discussion

The analysis of the oral production data shows that labels containing identical consonants are more accurately learned than those containing consonants that differ in place and manner, and that this learning advantage arises from a better retention of the repeated consonants. Mean edit distance between the target and the recalled form was significantly lower (i.e., more accurate) in the production of c-rep words than that of no-rep and v-rep words. Furthermore, when considering the accuracy with which consonants on the one hand and vowels on the other were reproduced, the only statistically significant effect was observed in the retention of consonants, which benefited from repetitions. By contrast, repetition of vowels did not facilitate recall accuracy, nor were vowels within a word better retained when they were identical rather than dissimilar.

These outcomes corroborate those from Ota et al. (2021; Experiment 2), in which participants exhibited better recall performance of orthographically presented words when they contained consonant letter repetitions, but not when they contained vowel letter repetitions. They are also in line with the extensive literature showing that speakers of a range of languages (e.g., English, French, Sesotho, Slovak, Spanish; cf. Nazzi & Cutler, 2019, p. 34) preferentially attend to consonants in lexical processing tasks. Thus, although repetitions within strings are known to confer a domain-general learning advantage, our findings indicate that when it comes to wordform learning, not all types of repetitions are made equal. Learning novel wordforms is made easier by the presence of identical consonants more so than that of identical vowels.

Our assumption behind this experiment was that wordforms with identical consonants are better learned due to the conjunction of a domain-general and a language-specific effect on memory: the advantage of string-internal repetition and the greater role of consonants over vowels in lexical distinctions. To minimize the possibility that the effects of consonantal and vocalic repetitions are due to differences in the typicality of such sequences in the participants’ native language rather than more general features of our lexical memory, we ensured that the novel word stimuli in the three conditions (i.e., c-reps, v-reps, and no-reps) were comparable in terms of English phonotactic probabilities and neighborhood density (see Table 1). However, there is some evidence that, in English, the overall probability of identical consonants or vowels cooccurring with one intervening segment is significantly less than expected by chance (Monaghan & Zuidema, 2015). This general tendency in the English lexicon might have affected the perceived phonotactic plausibility of our stimuli. Furthermore, in labeling tasks, English speakers have been shown to disprefer novel words with a string repetition (e.g., *slaflaf*) over those without such a repetition (e.g., *slafmak*), unless there is a morpho-semantic motivation (e.g., plurality) for the repetition (Berent et al., 2016; see also psycholinguistic evidence that English speakers are biased against words containing close cooccurrences of identical consonants: e.g., Coetzee, 2008). None of these studies reveal any difference in the extent to which consonant and vowel are underrepresented or avoided, but they do raise the possibility that our participants detected different levels of wellformedness or naturalness in c-reps, v-reps, and no-reps. For example, c-reps might have been perceived to be less phonotactically plausible or less wordlike than no-reps and v-reps. Given findings indicating that, under some circumstances, novel words with low phonotactic probabilities are better learned than those with high phonotactic probabilities (e.g., Storkel et al., 2006; Storkel & Lee, 2011), such differences could account for the outcomes of the recall results.

To address this issue, we carried out a two-alternative forced-choice experiment using the stimuli from Experiment 1 as possible names for novel objects. Thus, the semantic context of the task was the same as in the first experiment, but instead of having to learn and recall novel words, participants in Experiment 2 were asked to decide which of two novel words was more suitable as a label for one of the unfamiliar objects. One of the alternatives was always a no-rep word and the other was either a c-rep or a v-rep word. If c-reps and v-reps differed in perceived wellformedness, we expect to find a difference between c-reps and v-reps in their preference relationship to no-reps.

## 3 Experiment 2: label preference task

### 3.1 Method

This experiment tested adult listeners’ preferences for the nonwords with consonant repetitions, vowel repetitions, or no segmental repetitions used in Experiment 1. The design of the experiment is similar to that of Berent et al. (2016), in which participants had to choose one of two orthographically presented words as a label for a novel object. In our experiment, however, the stimuli were presented auditorily. As with Experiment 1, it was run online using the platform Testable.

#### 3.1.1 Participants

Participants, recruited via Prolific (www.prolific.co), were 36 adult native speakers of English with an approval rate of 95% or higher and a minimum of 50 submissions. They comprised 27 females and 9 males, ranging in age from 19 to 60 years (*M* = 34.0 years). They were self-reported monolingual native speakers of English, born in one of six English-speaking countries: U.K. (*n* = 26), U.S.A. (*n* = 6), Australia (*n* = 1), Canada (*n* = 1), Ireland (*n* = 1), and South Africa (*n* = 1). All of them were residents in their country of origin, except for one U.S. national currently living in the U.K.

Participants were asked to list any foreign languages they had studied. These included French (*n* = 20), German (*n* = 12), Spanish (*n* = 7), Italian (*n* = 3), British Sign Language (*n* = 1), Gujarati (*n* = 1), Irish (*n* = 1), Korean (*n* = 1), Mandarin Chinese (*n* = 1), Polish (*n* = 1), Russian (*n* = 1), Scots Gaelic (*n* = 1), and Welsh (*n* = 1). Participants received a compensation of £2 for completing the experiment, which took on average 7 min and 11 s.

#### 3.1.2 Materials

##### 3.1.2.1 Unfamiliar objects

These were the same as employed in Experiment 1 and can be seen in Appendix A. None of the objects was suggestive of reduplicative semantics (e.g., plurality, repetition, or intensity), which are known to attract responses in favor of sound repetitions (see Berent et al., 2016).

##### 3.1.2.2 Novel words

The 24 triplets of critical items and 12 fillers described in the Method section of Experiment 1 were also used in this experiment, as well as an additional 12 fillers—half of which were CVCVC words, and the other half, CVCVCV words.

#### 3.1.3 Procedure

The experiment consisted of 36 trials. At the beginning of each trial, a photograph of an unknown object (approximately 350 × 350 pixels in size) was presented in the middle of the screen. Two auditory words were played with a 1200-ms interstimulus interval. Participants were asked to select, by pressing the button “1” or “2,” which of the two words sounded like the more suitable name for that object. The order of the buttons was fixed (1 = left, 2 = right).

In 24 of the trials, the stimuli were two critical items and in the other 12, filler pairs. For the critical trials, the items of the 24 triplets were organized in pairs across two counterbalanced lists (see Appendix C), such that participants heard either a no-rep word presented alongside its c-rep counterpart (e.g., *fito* vs. *fifo*), or a no-rep word and its v-rep counterpart (e.g., *fito* vs. *fiti*). The two wordlists, containing an equal number of no-rep/c-rep and no-rep/v-rep pairs, were crossed with two randomly determined object mappings—thus reducing potential sound symbolism effects—to generate 4 stimulus combination lists, to which participants were evenly assigned.

The trial order was randomized within two blocks, each composed of 12 critical trials and 6 filler trials. In half of the critical trials, the no-rep word was played first, whereas in the other half, it was played after its v-rep or c-rep counterpart.

### 3.2 Results

For each trial, a selection for the label with a repetition (i.e., c-rep or v-rep) was coded as 1 and a selection for no-rep was coded as 0. Trials with reaction times of under 250 ms or greater than 2.5 standard deviations above the mean (4,897 ms) were deemed outliers and excluded (8 of 1,296 observations).

**Figure 4.**
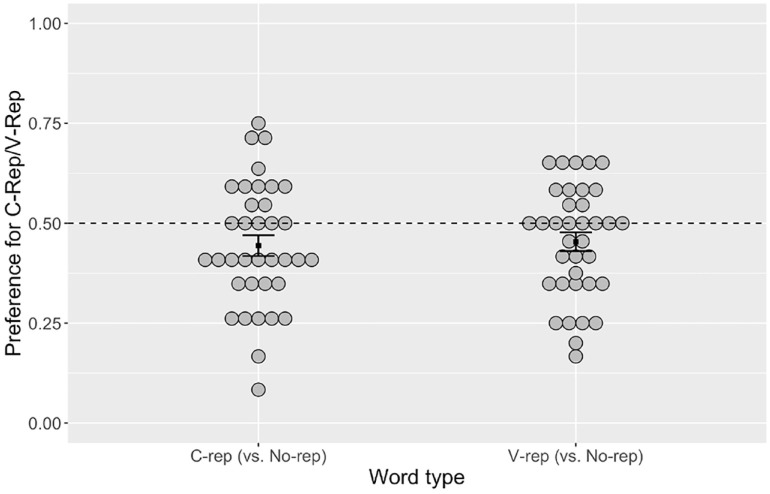
Proportion of Choice for Labels with Repetitions. *Note.* Gray circles represent participant means. Square points represent condition means. Error bars show ±1 standard error of means. Dotted line indicates chance level (0.5).

The results are illustrated in Figure 4. Values above 0.5 indicate a preference for c-reps/v-reps, and values below 0.5, a preference for no-reps. Group means were lower than 0.5 for both c-reps (0.442) and v-reps (0.453), showing that both repeated forms were dispreferred against no-reps.

A mixed-effects logistic regression model was carried out using the glmer function in R, with participants and item-pairs as random effects (Table 6). Word type was referenced to c-rep and dummy coded for v-rep. The results showed that the intercept was significantly below 0 (chance, in logit), confirming that novel words with segmental repetitions were generally dispreferred against words containing no segmental repetitions. There was no statistically significant effect of word type. In other words, there was no evidence that v-reps were either more or less favored than c-reps when compared with no-reps.

**Table 6.** Fixed Factor Coefficients: Preference for Words With Repetitions (C-Rep and V-Rep) Against No Repetition (No-Rep).

	β	*SE*	*z*	*z*	*p*
Intercept	−0.244	0.112	105.377	−2.190	<.029*
Word type	0.055	0.141	68.824	0.392	.695

* *p* < .05.

### 3.3 Discussion

The purpose of this follow-up experiment was to examine an alternative account of the results in Experiment 1. Although we ensured that the three word types (i.e., c-reps, v-reps, and no-reps) in Experiment 1 were similar in terms of the phonotactic probabilities and familiarity rating of the individual items, there remained a possibility that our participants’ perception of the novel words was affected by a different degree to which c-rep and v-rep-type words are generally underrepresented or considered wordlike in English. Under this scenario, we expect English speakers to show different levels of preference or dispreference for our c-rep stimuli and v-rep stimuli in comparison to no-reps.

Our results showed that when subjected to a label selection task using the same unfamiliar objects, both c-reps and v-reps were dispreferred against no-reps, and no difference was found between c-reps and v-reps in this regard. These outcomes are compatible with the observation that both c-rep and v-rep-type sequences are less common than no-rep-type sequences in the English lexicon (Monaghan & Zuidema, 2015) and replicate previous findings that demonstrate avoidance of consonant cooccurrences by speakers of English (Berent et al., 2016; Coetzee, 2008), Hebrew (Berent & Shimron, 1997), and Dutch (Boll-Avetisyan & Kager, 2014, 2016). Our experiment indicates that this effect may also apply to vowel repetition, at least in the case of English listeners.

However, the results of Experiment 2 in themselves cannot explain the findings from Experiment 1. Although we cannot rule out the possibility that there is an underlying difference in preference between c-reps and v-reps, the miniscule magnitude of estimated effect size (an odds ratio of 1.005 in favor of v-rep) makes it highly unlikely for it to be the cause of the robust contrast between c-reps and v-reps in Experiment 1. More importantly, if the results of Experiment 1 were due to variance in perceived wordlikeness of the novel words, we would expect to find a difference in recall accuracy between v-reps and no-reps since, like c-reps, v-reps are also dispreferred against no-reps as labels. This was not the case. All in all, these results do not support the possibility that the better recall accuracy in c-reps in Experiment 1 is due to stimulus characteristics that are extraneous to the effects of consonant repetitions in wordform learning. The advantage in learning c-rep words over no-rep words is therefore best interpreted as a reflection of a learning bias for wordforms containing repeated consonants.

## 4 General discussion

Are there language-independent biases that constrain the learning of new wordforms? To our knowledge, this question has not been extensively investigated beyond the effects of some basic characteristics such as the overall length of the word. Here, we attempted to expand our understanding of this issue by focusing on the effects of segmental repetitions within the word to be learned. Such effects can be predicted on the basis of cross-domain findings of memory facilitation from adjacent or near-adjacent repetition of elements in a string. At the same time, the advantage conferred by segmental repetitions is also likely to be conditioned by a well-known language-specific asymmetry in the role of consonants and vowels in lexical contrasts and lexical learning. These two lines of reasoning led us to the predictions that recall of recently encountered novel words would be facilitated by the presence of identical segments and that this facilitation would be greater in words with repeated consonants than those with repeated vowels.

These predictions were confirmed by our experiments. Experiment 1 demonstrated that English speakers trained on novel CVCV words mapped onto unfamiliar objects were better at recalling the wordforms corresponding to the objects when the novel word contained two identical consonants but no identical vowels (“c-rep”), compared with when the word contained no identical segments (“no-rep”), or two identical vowels but no identical consonants (“v-rep”). Our analysis also showed that words with consonant repetitions were better recalled than the others because learners specifically remembered the consonants more accurately when they were repeated. No such facilitation could be observed in repeated vowels. Experiment 2 was carried out to address the possibility that these outcomes reflect perceived differences in the wordlikeness or wellformedness between the three types of novel words. The same novel words and objects used in Experiment 1 were used in a forced-choice labeling task as a way to measure English speakers’ preference for c-reps and v-reps against no-reps. The results yielded no measurable difference between c-reps and v-reps, but a dispreference for both c-reps and v-reps over no-reps as novel labels. These outcomes indicate that the results of Experiment 1 cannot be explained away by appealing to the notion that c-rep words were thought to be more (or less) plausible than v-rep words as made-up words. Furthermore, the results corroborate a similar advantage of repeated consonant letters over repeated vowel letters in orthographic word learning that is reported by Ota et al. (2021). Given the results of the current study, the effects shown in orthographic word learning are likely to be a reflection of the phonological difference between consonants and vowels, which underlies the processing of letters that correspond to consonants and vowels. Taken together, our findings support the hypothesis that novel wordforms are retained better when they contain repeated segments, and that this effect is at least stronger in consonants than in vowels, or, possibly, unique to consonants.

To the extent that facilitation of learning from string-internal repetitions is also reported outside the domain of language, the consonant repetition effect we found here is best understood as a manifestation of a domain-general cognitive bias, albeit one that is conditioned by language-specific properties as it does not seem to apply across the board to all types of segments. In the “Introduction” section, we stated that the presence of such a bias in wordform learning raises at least three broader issues. In the paragraphs below, we revisit each of these points in light of the outcomes of our experiments.

A potential implication of a general learning bias for wordforms is that it may partially explain why we find some phonotactic effects on word learning. Suppose wordforms with a certain phonotactic configuration are inherently easier to learn due to a language-independent bias. Through cumulative diachronic changes responding to pressure from such bias, this may cause the proportion of words with that particular configuration to increase in the lexicon. Could this be the case with segmental repetition? In other words, are consonantal repetitions phonotactically favored across languages? At least on the surface, the answer is no. There is evidence in several languages, including English, that cooccurrence of identical consonants separated by a vowel is less frequent than expected by chance (Monaghan & Zuidema, 2015). In English, there are also specific contexts where identical noncoronal consonants are unattested (e.g., s_V_, as in *spap, *skak; Coetzee, 2008). By this measure, the form that is favored by learning is not phonotactically more frequent. This observation is also consistent with the results of our second experiment, which shows that compared with wordforms consisting of different segments, wordforms with segmental repetitions are dispreferred as labels, an effect that may be related to the generally low phonotactic probability of segmental repetitions in English.

However, a learning bias for wordforms with consonant repetitions may have a different type of impact on the general shape of words across languages. As noted in the “Introduction” section, one phonological phenomenon that could be rooted in this learning bias is what is known as the “identity effect.” The identity effect refers to a tendency for languages to allow identical consonants to escape restrictions on cooccurrences of feature-sharing consonants. For example, avoidance of consonants sharing a major place in a CVC sequence (e.g., *pVm) often underapplies to identical consonants (e.g., pVp), even though they meet the featural description of the banned sequence. The classic autosegmental explanation of this reversal is that identical consonants are not subject to phonological restrictions on two feature-sharing segments because they are underlyingly a single featural representation linked to two positions in the string (Goldsmith, 1976; McCarthy, 1988). Another proposal made in the literature is that words containing identical consonants are not underattested as much as those containing nonidentical feature-sharing consonants because they are made more distinct from other words in the lexicon by virtue of the underattestation of nonidentical but similar consonants (Graff, 2012). The learning advantage found for identical consonants here offers an alternative account for the identity effect. That is, the constraint on feature-sharing consonants may underapply to identical consonants because the benefits of having more learnable structures in the lexicon counteract the pressure of minimizing confusability between lexical items. This interpretation of the relationship between the identity effect and the advantage of consonant repetition in word learning raises the possibility that word-learning biases may be contributing to some cross-linguistically common phonological patterns.

A learning bias for consonant repetitions may also play a role in another well-attested phonological phenomenon, albeit in developing language systems. Across languages, young children frequently produce non-target-like wordforms in which two consonants separated by a vowel assimilate in major place (e.g., *doggy* produced as [gɔgi]; *coat* produced as [kok]; see Levelt, 2011 for an overview). Dubbed “child consonant harmony,” this phenomenon has attracted a great amount of interest in phonological analysis of children’s word production (Fikkert & Levelt, 2008; Goad, 1997; Pater & Werle, 2003; Smith, 1973; Vihman, 1978). One reason for this attention in the literature is that child consonant harmony lacks an adult analog—if anything, there is an opposite tendency across (adult) languages to avoid cooccurrences of consonants sharing the same major place. As a consequence, child consonant harmony resists a straightforward account built on standard analyses of place assimilation in adult phonology, leading some to propose that child consonant harmony is a form of phonologized speech errors (Hansson, 2001) or a product of “immature” phonological representations in which place features are associated with the entire word rather than individual segments (Fikkert & Levelt, 2008).

The results of our study raise another possibility. If repeated consonants facilitate wordform learning because they allow memory compression, they may also offer a fallback structure when the learner’s memory is taxed, as might be the case for children learning new words. Forms such as [gɔg] and [gɔgi] for *dog*/*doggie* could therefore be transitory forms of words that pose representational challenges for children due to the presence of consonants with different major places (see Vihman et al., 2023 for a related argument). This account is consistent with the observation that the majority of reported cases of child consonant harmony actually involve identical rather than feature-sharing consonants, especially when one takes into consideration that voicing contrasts in children’s production cannot be reliably detected by an adult listener (e.g., what is transcribed as [gʌk] may be /gʌg/ or /kʌk/; Macken & Barton, 1980). Further support for this interpretation comes from experimental evidence that older (4- to 9-year-old) children frequently insert repeated consonants in unsuccessfully remembered words (e.g., [tuːdu], [kukuː], [kəɡuː] for *tudu*; Aitchison & Chiat, 1981).

To test the viability of this argument, we conducted an exploratory analysis of the errors produced by our participants in response to no-rep targets. The mean proportion of misremembered no-reps produced with identical consonants (e.g., *kika* for *kifa*) was 17%, which was significantly higher than a simulated chance level of 9% (an estimate we obtained by randomly recombining all the consonants that participants used in their recalls, repeated over 10,000 times). These results suggest that even adults tend to repeat a consonant when they fail to recall a newly learned word in full, a pattern that may underlie child consonant harmony.

Our hypothesis that repetition of the same segments aids learning of words is motivated on the basis of similar effects from other types of string learning (e.g., numbers, visual patterns). If this effect is grounded in such domain-general memory bias, we expect it to show other characteristics of intraserial repetition on learning that have been observed across domains. One well-established finding in serial memory of letter and number sequences is that when the repeated elements are intervened by two or more other elements, accurate recall is inhibited, especially on the second occurrence (the Ranschburg effect: Crowder, 1968; Henson, 1998; Jahnke, 1969; Kahana & Jacobs, 2000). If the consonant repetition facilitation reported here shares a domain-general memory basis with recall of alphanumeric strings, we should expect to see similar effects. For example, the second /p/ in *pagipo* would be less accurately recalled than the /t/ in *pagito*. This type of prediction can be tested in future work to further probe the domain-general foundation of repetition facilitation in wordform learning.

An aspect of our predictions that is based on language-specific features, rather than a domain-general bias, is that repetition effects should be stronger for consonants than vowels. This was based on the model and related findings of the C/V hypothesis, which states that consonants play a larger role than vowels in lexical distinctions (Nespor et al., 2003). Our results are in line with this idea. It should be noted, however, that the preponderant role of consonants in establishing lexical contrasts might not be a universal phenomenon, but rather a function of specific languages’ phonemic inventories. Indeed, studies on lexical processing by learners and speakers of a language with more vowels than consonants (Danish: Højen & Nazzi, 2016) or of languages with lexical tone, which is mainly carried by vowels (Cantonese: Chen et al., 2021; Gómez et al., 2018; Mandarin: Wewalaarachchi et al., 2017; Wewalaarachchi & Singh, 2020; Wiener & Turnbull, 2016), found either no bias for one type of segment, or a vowel bias. Relatedly, a corpus analysis of Mandarin by Tong et al. (2008) indicates that in this language, vowels have the highest informational value in distinguishing phonological neighbors (although see Oh et al., 2015 for a different outcome). Another potential source of the C/V asymmetry that may stem from the characteristics of individual languages is the effect of dialectal variation (Floccia et al., 2009). In English, for example, phonological differences between dialects involve more variation in vowels than consonants (Wells, 1982). This tendency may lead adult speakers of English and other such languages to pay more attention to consonants when processing words. Future research could examine whether the consonant repetition effect on word learning found in Experiment 1 extends to languages with more vowels than consonants and/or with lexical tone, or whether in these languages repeated vowels prove more advantageous than repeated consonants.

The ratio between consonants and vowels in our experiment may likewise have had an effect on the way participants responded to the task. On the one hand, if participants were keeping track of the ratio of consonants (nine) and vowels (five) in the inventory of this artificial language, they might have noticed that the proportion of critical items in which consonants are repeated (four out of 18, or about 0.22) was higher than what can be expected by chance from a random combination of nine consonants (approximately 0.11). By contrast, the proportion of critical items in which vowels were repeated (also 0.22) was about the same as what is expected by chance (0.20). This might have drawn participants’ attention to the repetition of consonants. On the other hand, the possible combinations of vowels (25) were much fewer than those of consonants (81), which was in favor of remembering the sequence of vowels and correctly guessing them by chance. We do not have enough information to assess how these two competing factors might have interacted with each other in relation to the contrasting outcomes for consonant versus vowel repetitions. This issue needs to be investigated in further work using words based on a more balanced segmental inventory.

The scope of the current study is limited by a number of other factors that we have decided to leave for future exploration. First, because all of our critical items had the structure CVCV, segment types (i.e., consonants versus vowels) were conflated with their positions. It is challenging to fully tease apart these factors with respect to syllable structure due to the inherent affinity between consonants and syllable onsets on the one hand and vowels and syllable nuclei on the other. However, as has been done by Creel et al. (2006), it is possible to control for word-internal positions by using stimuli such as VCVC, where the word-initial position is occupied by a vowel and the word-final position by a consonant, in contrast to the opposite positioning in CVCV words. A related issue in relation to the CVCV structure of the critical stimuli is that the two consonants are in the same within-syllable position (i.e., the onset) but in separate syllables. It may be that consonant repetition shows a different effect when the consonants are in the same syllable but in different within-syllable positions (e.g., CVC). Furthermore, syllable structure is likely to interact with how intervening materials are interpreted. For example, the repetition of /p/ in the onset might have a different effect on the learning of words like *pipa* and *pimpa*. All of these questions need to be explored further with the use of different novel word structures. Second, to measure the phonological distance between target nonwords and participants’ recalled forms, we used Levenshtein distance, a method that is widely employed in psycholinguistic studies, but which has some shortcomings. Chief among them is that different types of deviance (e.g., substitution, deletion, and addition) are treated equally and so are different types of substitutions. This means that, for a target example /pupe/, recalled forms such as /pu/, /tupi/ and /fufe/ were treated as equally deviant, when a more phonologically sensitive measure would have differentiated them in terms of their proximity to the target. Although deciding how to measure the phonological distance between two strings is not a trivial issue (Heeringa et al., 2009; Kondrak, 2002; Nerbonne & Heeringa, 2001; Sanders & Chin, 2009), it is possible that a different type of measurement of the recall errors may uncover more subtle effects of segmental repetitions. Fourth, by focusing exclusively on segmental repetitions, we have not considered how their effects on word learning might compare to that of other kinds of word-internal repetitions, such as syllable reduplication. As mentioned in the “Introduction” section, repeated syllables have a facilitatory effect on infants’ segmentation and recall of novel words containing them (Ota & Skarabela, 2016, 2018). Whether syllable reduplication could have a similar facilitatory effect in adult word learning, and if so, whether such effect would be stronger than that of consonant repetitions, remains to be seen. Finally, our investigation targeted only one phase of learning, which resulted from a short training exposure to the novel words. However, memory of novel words is known to shift in characteristics from the initial point of exposure, early encoding and long-term consolidation (Gaskell & Dumay, 2003; Storkel, 2001). How the effects of word-internal segmental repetitions on word learning change over the course of this process is another issue that needs to be addressed in future work.

This study set out to examine the idea that words with certain shapes are easier to learn, not because of their language-specific phonotactic or lexical properties, but because of general structural characteristics that make them inherently more learnable. We specifically targeted the role of segmental repetitions within words, based on previous across-domain findings that learning of strings is facilitated by the repetition of identical elements in proximity. The results of our study with English speakers confirm that this domain-general trait generalizes to novel word learning, but the effects were conditioned by segment types, with the repetition advantage found only in consonants and not in vowels. It remains to be seen if these findings can be replicated in speakers of other languages, but the overall outcome of our study suggests that the notion of a language-independent bias on word learnability—something that has been studied so far in limited contexts, such as word length—can be usefully explored in research on word learning. As we have hopefully demonstrated, the presence and effects of such a factor on wordform learning have important implications not only on how words are learned but also, potentially, on how general phonological properties of the lexicon are shaped across languages.

## Figures and Tables

**Figure:**
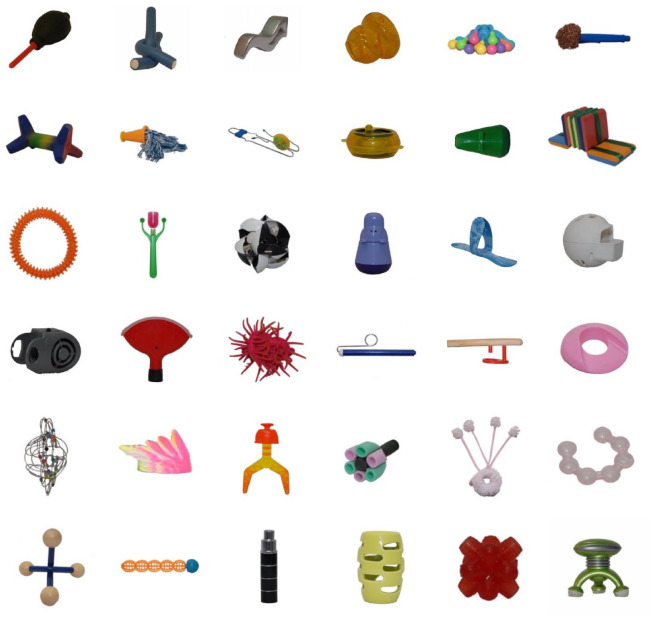
Unfamiliar Objects Used in the Experiment. Source: NOUN database (Horst & Hout, 2016).

## Appendix B

**Table:** Lexicons Used in Experiment 1.

	Lexicon A	Lexicon B	Lexicon C	Lexicon D	Lexicon E	Lexicon F
C-rep	fofi	fifo	sase	suse	nane	fefa
	ʧaʧi	tita	mema	lali	kuke	kika
	tute	memu	noni	mimo	ʧeʧa	nune
	lilo	kake	lelu	ʧaʧe	fafi	sesu
V-rep	nupu	suku	fala	ʧafa	tifi	meke
	lapa	naka	foʧo	fene	meʧe	ʧama
	ʧepe	lefe	sepe	fiti	nopo	tumu
	kuʧu	mili	kifi	kala	sama	lipi
No-rep	sepu	kifa	meku	nupe	fito	foʧi
	fena	nopi	nake	kuʧe	suke	lapi
	ʧame	same	tume	tifa	kale	lefu
	fali	meʧa	ʧepa	lipo	ʧafi	milo
Fillers	mapil	ʧofas	mapil	ʧofas	mapil	ʧofas
	nepas	fales	nepas	fales	nepas	fales
	sopek	kisen	sopek	kisen	sopek	kisen
	ʧisofa	safemi	ʧisofa	safemi	ʧisofa	safemi
	lutefa	tikoʧo	lutefa	tikoʧo	lutefa	tikoʧo
	nupale	pisole	nupale	pisole	nupale	pisole

## Appendix C

**Table:** Lists of Stimuli Used in Experiment 2 (Critical Item Pairs).

List A		List B	
sepu	sesu	sepu	sepe
nopi	nopo	nopi	noni
lipo	lilo	lipo	lipi
ʧepa	ʧepe	ʧepa	ʧeʧa
fito	fifo	fito	fiti
tume	tumu	tume	tute
kifa	kika	kifa	kifi
suke	suku	suke	suse
meku	memu	meku	meke
nake	naka	nake	nane
kale	kake	kale	kala
kuʧe	kuʧu	kuʧe	kuke
fena	fefa	fena	fene
lefu	lefe	lefu	lelu
ʧafi	ʧaʧi	ʧafi	ʧafa
same	sama	same	sase
ʧame	ʧaʧe	ʧame	ʧama
milo	mili	milo	mimo
fali	fafi	fali	fala
meʧa	meʧe	meʧa	mema
tifa	tita	tifa	tifi
nupe	nupu	nupe	nune
foʧi	fofi	foʧi	foʧo
lapi	lapa	lapi	lali

**Table:** Lists of Stimuli Used in Experiment 2 (Filler Pairs).

List A		List B	
fales	tuʧak	fales	tuʧak
mapil	lemos	mapil	lemos
tupas	kisen	tupas	kisen
fumil	ʧofas	fumil	ʧofas
taʧol	sopek	taʧol	sopek
futen	nepas	futen	nepas
safemi	tupano	safemi	tupano
makino	lutefa	makino	lutefa
kofemo	ʧisofa	kofemo	ʧisofa
solima	nupale	solima	nupale
tikoʧo	lukema	tikoʧo	lukema
pisole	fusino	pisole	fusino

## Notes

1. We use phonetic symbols to transcribe our stimulus words (e.g., fifo = [fifo], not [faɪfoʊ]).

2. We also measured accuracy using a binary distinction between a perfect match (1) and any form of mismatch (0). However, as the task was relatively difficult, this coarse-grained measure resulted in data with a floor effect that could not be subjected to reliable statistical analyses.

3. The model reported here had the structure: edit.distance ~ word.type + perceptual.error + (1|participant) + (1|item). Although the model with random slopes for participants yielded a singular fit, its outcomes are comparable to the reduced model, with a significant effect for no-reps (β = 0.099, *df* = 68.5, *p* = .033) and v-reps (β = 0.120, *df* = 71.3, *p* = .013).

4. The models reported here had the structure: consonant.edit.distance ~ word.type + perceptual.error + (1|participant) + (1|item) and vowel.edit.distance ~ word.type + perceptual.error + (1|participant) + (1|item). Although the models with random by-participant slopes for word.type and perceptual.error yielded singular fits, their outcomes were comparable to the reduced models, with significant effects for no-reps (β = 0.128, *df* = 67.87, *p* = .020), v-reps (β = 0.145, *df* = 70.97, *p* = .011), and perceptual error (β = 0.052, *df* = 61.74, *p* = .028) for the consonant-only analysis, but no effects of word type or perceptual error for the vowel-only analysis.
